# Heart Failure after Laboratory Confirmed Influenza Infection (FLU-HF)

**DOI:** 10.5334/gh.1125

**Published:** 2022-06-23

**Authors:** Phyllis Sin, Muhammad Siddiqui, Rashell Wozniak, Idris Bare, Jessica Minion, Stephen Sanche, Jacob Udell, Andrea Lavoie, Payam Dehghani

**Affiliations:** 1 University of Manitoba, The Max Rady College of Medicine, Winnipeg, MB, CA; 2 Saskatchewan Health Authority, Research Department, Regina, SK, CA; 3 University of Alberta, Edmonton, AB, CA; 4 University of Saskatchewan, College of Medicine, Saskatoon, SK, CA; 5 University of Saskatchewan, College of Medicine, Regina, SK, CA; 6 University of Toronto, Faculty of Medicine, Toronto, ON, CA

**Keywords:** Influenza, respiratory infection, heart failure, hospitalization

## Abstract

**Background:**

Influenza has been shown to exacerbate heart failure (HF). Importantly, no study to date has examined the relationship between HF hospitalizations (HFH) with laboratory confirmed influenza infections. This study evaluated the association between laboratory confirmed influenza infection and HFH in the two largest hospitals in Saskatchewan, Canada.

**Methods:**

We used a retrospective self-controlled case series design to evaluate the association between laboratory-confirmed influenza infection and HFH. We compared the incidence ratio for HFH during the influenza risk interval with the control interval. We defined the influenza risk interval as the seven days after a laboratory confirmed influenza result and the control interval as one year before and after the risk interval.

**Results:**

We identified 114 HFH that occurred within one year before and after a positive test result for influenza between April 1, 2010, and April 30, 2018. Of these, 28 (28 admissions per week) occurred during the risk interval and 86 (0.853 admissions per week) occurred during the control interval. The incidence ratio of a HFH during the risk interval as compared with the control interval was 33.53 (95% confidence interval [CI], 21.89 to 51.36). A decline in incidence was observed after day seven; between days 8 to 14 and 14 to 28 incidence ratios was 0.91 (95% CI, 0.13 to 6.52) and 0.91 (95% CI, 0.22 to 3.68) respectively.

**Conclusion:**

We have observed a significant association between acute influenza infection and HFH. However, further research with a larger sample size and involving a multicenter setting is warranted.

**Highlights:**

- Influenza may contribute and exacerbate heart failure events especially during annual influenza season.
- Early identification of influenza among patients with heart failure, could lead to earlier treatment with antiviral medication, reduce unnecessary antibiotic use, and tail off the morbidity and mortality.
- In this study, despite our efficient study design, our sample size was limited to only the two largest hospitals in the province, possibly excluding a significant population in remote areas.

## Introduction

Influenza epidemics are associated with substantial morbidity and mortality, with peak activity during winter months [1]. The World Health Organization estimates three to five million cases of influenza and approximately 300,000 to 650,000 deaths worldwide are attributable to influenza annually. Influenza can trigger acute cardiovascular events such as myocardial infarction and death [2]. In North America, heart failure hospitalizations (HFHs) peak during the winter influenza season and are lowest during the summer [3]. This is particularly relevant in Saskatchewan, Canada, where the annual influenza rates are disproportionately high in comparison to provinces with similar climate and population [4]. Pneumococcal and influenza respiratory infections have been known to exacerbate HF resulting in prolonged hospitalizations. This is due to a cascade of effects including a sudden onset of fever, tachycardia, dehydration, hypoxemia, endothelial dysfunction, hypercoagulation and bolus secretion of pro-inflammatory mediators [5]. This is further corroborated by reductions in HFHs and death with influenza vaccination [3]. In fact, annual influenza vaccination in patients with acute and chronic HF is an essential topic for patient education by the European Society of Cardiology and the Canadian Cardiovascular Society [6,7].

It is known that acute myocardial infarction has been tied to influenza; however, there is a paucity of studies demonstrating a similar relationship with HF [2]. Most available data on HFHs are limited to small samples, single centers, or derived from observations of patients enrolled in clinical trials, which have select enrollment criteria [8]. No study to date has examined the relationship between HFHs with laboratory confirmed influenza infections.

Given the large gap in the literature and the potential implications of preventing HF morbidity due to hospitalizations, we propose a retrospective self-controlled case-series design to evaluate the association between laboratory confirmed influenza infection and HFH at two major hospitals in Saskatchewan. The primary objective is to examine whether HFHs correlate with laboratory confirmed influenza.

## Materials and methods

**Figure 1:**
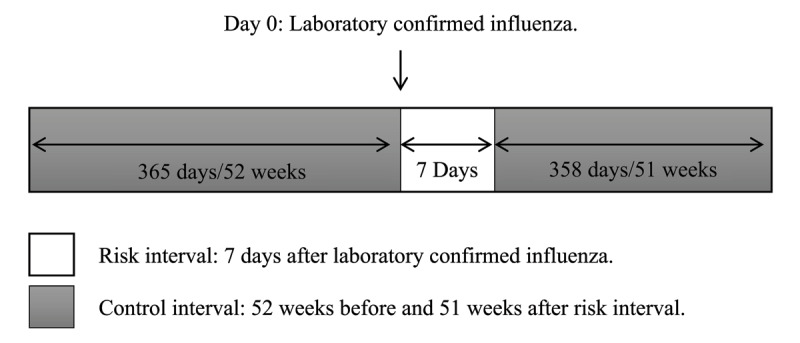
**Influenza Infection Timeline.** Influenza risk interval is defined as the 7 days after laboratory confirmed influenza. Influenza control interval is defined as the 365 days or 52 weeks before and 358 days or 51 weeks after the risk interval.

This retrospective population-based study with a self-controlled case-series evaluated the association between laboratory confirmed influenza infection and HFHs. The incidence ratio for HFHs during the influenza risk interval was compared with the control interval (Figure 1).

This study was funded by the University of Saskatchewan College of Medicine Research Award 2018. It was reviewed and approved on ethical grounds by the research ethics board of the former Regina Qu’Appelle Health Region, Regina, SK, Canada (REB/18-70). For this study, it was impracticable for informed consent to be obtained. As such, a waiver of informed consent was granted by the research ethics board. The study was performed in accordance with the relevant guidelines and regulations as outlined by the former Regina Research Ethics Board, Saskatchewan Health Authority, Regina, SK, Canada.

## Study design

**Figure 2:**
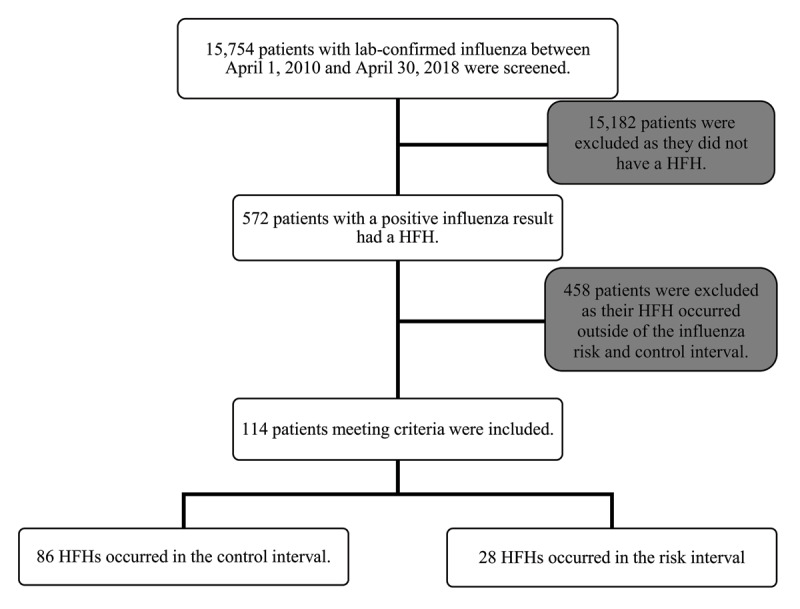
**Flow of Study Participants.** Influenza positive patients stratified based on their heart failure hospitalization (HFH) during the influenza risk interval compared to the control interval.

We screened all subjects with positive influenza respiratory specimens between April 1, 2010 and April 30, 2018 from the Roy Romanow Provincial Laboratory in Saskatchewan, Canada. Of these cases, we identified those that were hospitalized with HF as the primary diagnosis at discharge ascertained from administrative data from Saskatchewan Health Authority health records. We used the following International Classification of Diseases 10 diagnostic codes: I50.0 (Congestive heart failure), I50.1 (Left ventricular failure) and I50.9 (Heart failure, unspecified). Based on the average influenza viral shedding period, we defined the influenza risk interval as the seven days after a respiratory specimen was confirmed by the laboratory to be positive for influenza [2,9]. We defined the influenza control interval as the 52 weeks before and 51 weeks after the risk interval (Figure 1) [2]. Further stratification allowed patients admitted with HF during the influenza risk interval to be compared with patients admitted with HF during the influenza control interval (Figure 2). Eligibility requirements at screening included an age of 18 years or older, and a HFH that occurred within 52 weeks before and after a positive influenza test between April 1, 2010, and April 30, 2018. Exclusion criteria included HFHs with acute coronary syndrome as a concurrent diagnosis, if the presence of HF could not be objectively determined using the above inclusion criteria, or if hospital records were incomplete. We restricted the analysis to the first event in an episode of care by excluding admissions within 30 days after a previous hospital discharge for HF for the same patient.

## Study procedures

Data about respiratory specimens were obtained from the Roy Romanow Provincial Laboratory. Consistent high-specificity laboratory methods (reverse-transcriptase polymerase chain reaction (PCR; monoplex or multiplex assays), viral culture, direct fluorescent antibody staining, and enzyme immunoassays) confirmed influenza infection. The following viruses were of interest to our study: Influenza A and Influenza B. Data collection including subject demographic information, comorbidities, medications, laboratory tests, and diagnostic imaging reports were conducted by an investigator familiar with the study protocol. The subject record documented the subject’s study identification, demographic information meeting the inclusion and exclusion criteria, discharge diagnostic codes and supporting clinical information. Pertinent clinical information was also included: echocardiogram showing ejection fraction (EF), current medications, comorbid conditions, elevated plasma B-type natriuretic peptide (BNP) level of at least 150 pg per milliliter or an N-terminal pro-BNP (NT-pro BNP) level ≥ 600 pg per milliliter [1,7].

## Statistical analysis

Incidence ratios were estimated with the use of a fixed-effects conditional Poisson regression model. In terms of sample size calculation, Musonda et al. (2006) described a formula where 97 participants would be needed to achieve 80% power [10]. Statistical analysis was performed using SPSS Statistics software (Version 22.0. Armonk, NY: IBM Corporation). The incidence ratio for HFHs during the influenza risk interval as compared to the control interval was determined using the ratio of the incidence rate in the risk interval group divided by the incidence rate in the control interval group [2]. The incidence rate was ascertained by dividing the number of HFHs by the number of weeks in that interval; for the risk interval, it would be one week and for the control interval, it would be 103 weeks. In addition to the primary analysis that defined the influenza risk interval as day one to seven after the index date, we also considered narrower risk intervals (day one to three) and alternative intervals (day eight to 14 and day 15 to 28). Chi-square test was used as a test of significance to compare differences between groups for categorical data. We performed analyses in subgroups defined according to age (≤65 years vs. >65 years), gender, virus type (influenza A [all subtypes] vs. B), history of ischemic heart disease and diabetes (yes vs. no). We evaluated the presence of interactions in these subgroups. Statistical significance would be set at p < 0.05.

## Results

We identified 15,754 subjects with positive influenza respiratory specimens between April 1, 2010 and April 30, 2018; 8188 were from the city of Regina and 7566 from Saskatoon. Of these, 572 patients (396 from Regina and 176 from Saskatoon) were admitted to hospital with HF as the primary diagnosis at discharge. We excluded 458 patients as their HFH occurred 52 weeks after their positive influenza result (Figure 2). Therefore, in this retrospective population-based analysis, 114 HFHs occurred within 52 weeks before and after a positive test result for influenza. The characteristics of the patients at baseline were balanced between the risk interval and control interval groups (Table 1). The mean age of the study population was 82.6 years (standard deviation 12.9) and 42.1% of the patients were female, of which 79.8% (n = 91) were from Regina and 20.2% (n = 23) were from Saskatoon. The mean EF was 46.11% ± 14.5 and mean BMI was 29.8 kg/m^2^ ± 9.2. The median BNP was 700.50 pg/mL. Majority of infections (83.3%) were due to influenza A which is consistent with Canadian epidemiologic data from that time period [4].

**Table 1:** Baseline Characteristics.


	TOTAL N = 114	RISK INTERVAL N = 28	CONTROL INTERVAL N = 86	P-VALUE

**Gender, n (%)**				0.16

Male	66 (57.9)	13 (46.4)	53 (61.6)	

Female	48 (42.1)	15 (53.6)	33 (38.4)	

**Coronary Artery Disease, n (%)**				0.51

No	79 (69.3)	18 (64.3)	61 (70.9)	

Yes	35 (30.7)	10 (35.7)	25 (29.1)	

**Hypertension, n (%)**				0.14

No	22 (19.3)	3 (10.7)	19 (22.1)	

Yes	92 (81.7)	25 (89.3)	67 (77.9)	

**Diabetes, n (%)**				0.28

No	63 (55.3)	13 (46.4)	50 (58.1)	

Yes	51 (44.7)	15 (53.6)	36 (41.9)	

**Dyslipidemia, n (%)**				0.26

No	90 (78.9)	20 (71.4)	70 (81.4)	

Yes	24 (21.1)	8 (28.6)	16 (18.6)	

**Cerebrovascular Disease, n (%)**				0.12

No	96 (84.2)	26 (92.9)	70 (81.4)	

Yes	18 (15.8)	2 (7.1)	16 (18.6)	

**Smoking, n (%)**				0.42

No	84 (73.7)	19 (67.9)	65 (75.6)	

Yes	30 (26.3)	9 (32.1)	21 (24.4)	

**Peripheral Vascular Disease, n (%)**				0.61

No	105 (92.1)	26 (92.9)	79 (91.9)	

Yes	9 (7.9)	2 (7.1)	7 (8.1)	

**Mean BMI (kg/m²) ± SD**	29.82 ± 9.2	27.74 ± 8.2	30.35 ± 9.5	0.35

**Mean EF (%) ± SD**	46.1 ± 14.5	47.2 ± 13.9	45.6 ± 14.9	0.72

**Median BNP (pg/mL) (IQR)**	700.50 (922)	729.69 (1135)	657 (773)	0.58


Twenty-eight HFHs occurred during the influenza risk interval and 86 HFHs occurred during the control interval. Specifically, there were 28 HFHs in the one week after a patient’s respiratory specimen was positive for influenza and only 0.835 HFHs occurred per week in the 52 weeks before and 51 weeks after the risk interval. Therefore, the incidence rate of the influenza risk interval was 28 because 28 HFHs occurred in one week, and the incidence rate in the control interval was 0.835 because 86 HFHs occurred in 103 weeks (Table 2).

**Table 2:** Incidence Rate and Ratio Sample Calculation.


INFLUENZA TIME INTERVAL	INCIDENCE RATE	INCIDENCE RATIO

Risk Interval	\frac{{28\;HFHs}}{{1\;week}} = 28\;HFH{\rm{/}}week	\frac{{28\;HFHs{\rm{/}}week}}{{0.835\;HFHs{\rm{/}}week}} = 33.53

Control Interval	\frac{{\left( {54 + 32} \right)HFHs}}{{\left( {52 + 51} \right)weeks}} = 0.835\;HFHs{\rm{/}}week	


**Figure 3:**
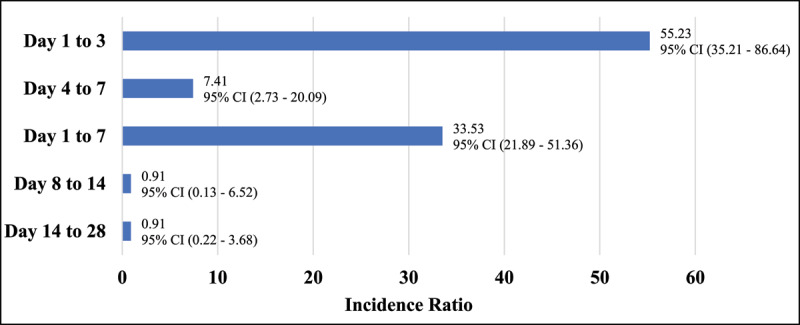
Incidence Ratios for Heart Failure Hospitalization According to the Days Following Laboratory-Confirmed Influenza Infection.

Consequently, the incidence ratio of HFH during the risk interval as compared with the control interval was 33.53 (95% CI, 21.89 to 51.36). In looking at the first three days of the risk interval, the incidence ratio for HFHs is even higher at 55.23 (95% CI, 35.21 – 86.64) (Figure 3). Evidently, the first few days following a positive influenza result is the driving factor behind the increased incidence ratio for HFHs during the influenza risk interval. A decline in incidence was observed after day seven; between day’s eight to 14 and 14 to 28 incidence ratios was 0.91 (95% CI, 0.13 to 6.52) and 0.91 (95% CI, 0.22 to 3.68) respectively (Figure 3).

In the subgroup analyses, an elevated incidence of HFHs after influenza infection was observed among adults older than 65 years of age but not for younger adults. However, the difference was not statistically significant (p = 0.23). The incidence ratios were higher for influenza B than for influenza A, but the interaction did not meet statistical significance (p = 0.17). The incidence of HFH was elevated in patients with a history of coronary artery disease and diabetes, but the interaction was not statistically significant (Table 3).

**Table 3:** Subgroup Analyses Comparing Incidence Ratios for Heart Failure Hospitalization after Laboratory-Confirmed Influenza Infection.


SUBGROUP	INCIDENCE RATIO (95% CONFIDENCE INTERVAL)	P-VALUE FOR INTERACTION

**Age**		0.23

≤65 years	55.46 (22.13–139.01)	

>65 years	29.63 (18.24–48.14)	

**Gender**		0.16

Male	25.26 (13.77–46.33)	

Female	46.82 (25.43–86.19)	

**Influenza Type**		0.17

Influenza A	29.23 (18.0–47.46)	

Influenza B	60.08 (23.65–152.60)	

**Coronary Artery Disease**		0.51

Yes	41.2 (19.79–85.78)	

No	30.39 (17.96–51.41)	

**Diabetes**		0.28

Yes	42.92 (23.49–78.39)	

No	26.78 (14.55–49.29)	


## Discussion

Our study aimed to review the impact of acute influenza on HF. This retrospective population-based study with a self-controlled case-series used laboratory confirmed influenza cases to assess the association with HFHs in the province of Saskatchewan in Canada. Similar studies focused solely on the association between influenza and acute coronary syndrome [2]; but none have studied the link between acute influenza infection and HFHs. As such, the primary objective was to examine whether HFHs in two major hospitals in Saskatchewan were correlated with laboratory confirmed influenza. We found that the incidence of HFHs was 33 times higher during the first week after laboratory confirmation of influenza infection compared to control interval (28 admissions per week vs. 1.7 admissions per week).

Heart failure (HF) is a chronic disease with no cure that is associated with significant morbidity and mortality. Currently, 600,000 Canadians live with HF and more than 26,000 residents above the age of 40 are reported to have HF in the province of Saskatchewan alone [11]. Heart failure hospitalizations (HFH) have increased annually across Canada, with 60,000 reported in 2013–2014 according to the Canadian Institute for Health Information [12]. Further, HF prevalence increases with age and it is also known that elderly HF patients are at highest risk for hospitalizations and developing complications from influenza infections [13]. Evidently, HF carries a significant burden to our healthcare landscape today.

Most of the literature looking at respiratory infections and cardiovascular disease focus on myocardial infarction or stroke risk. Presently, a causative association between influenza infection and HF exacerbation has not been demonstrated [14]. Influenza infection may induce direct myocardial dysfunction through immune mediated inflammation proven by histologically evident myocarditis and necrosis after influenza-related deaths [15]. This association is the result of pro-inflammatory cytokines produced during an influenza infection that accelerate atherogenesis and impair cardiac inotropy, resulting in adverse myocardial remodeling [16]. In terms of HF pathophysiology, high metabolic demands, and potent inflammatory agents activated by influenza infection may indirectly suppress myocardial function leading to either new onset HF or acute decompensation of chronic HF [15]. Further, influenza mediated changes in cardio-renal function may exaggerate fluid shifts resulting in volume overload and hence HF progression, or decompensation [15]. Notably, no studies have shed light on the clinical relevance of pathophysiologic interference between HF and influenza infection.

Influenza may trigger HF exacerbations requiring hospitalization in patients with HF. It is known that HF is an independent prognostic factor for influenza associated hospitalizations or death [3]. An analysis of OPTIMIZE-HF (the Organized Program to Initiate Lifesaving Treatment in Hospitalized Patients with Heart Failure) revealed that pneumonia or other respiratory processes precipitated 15.3% of HFHs and increased the risk for in-hospital mortality by 60% [8]. This is particularly relevant for our study as the incidence of HFHs was 33 times higher immediately following an influenza respiratory infection. Further, the mean age of our population was 83 years old, reflecting that HF patients are typically older and possess limited cardiac and respiratory reserve to tolerate infection-induced cardiac compromise [14]. Interestingly, HF decompensation occurred most commonly during the first week of influenza infection; the majority within the first three days [15]. This was also seen in our results as most HFHs occurred during the narrower influenza risk intervals, specifically day one to three (Figure 3). Further, numerous observational studies have shown an elevated risk for acute cardiovascular events including myocardial infarction within the first few days of influenza infection [17,18]. Specifically, parallel fluctuations of HFHs and seasonal influenza infections have been reported [18,19]. More recently, in a database of over 55,000 patients hospitalized for HF with and without influenza, Panhwar et al. reported increased rates of in-hospital mortality, adverse clinical outcomes, and prolonged length of stay among those with influenza compared with individuals without influenza [20]. Not only are HF patients being hospitalized, but their course in hospital is associated with significant morbidity and mortality.

Clinically, influenza is on the differential for causing HF exacerbation [8,14]. Perhaps prophylactic measures against influenza should be a consideration for HF patients, especially in the elderly given their trend towards an increased incidence of HFHs. Further information regarding the clinical significance of identifying acute influenza in HF patients is needed as treatment of acute influenza infections should be considered.

Our study had limitations. Firstly, despite our efficient study design, our sample size was limited to only the two largest hospitals in the province of Saskatchewan possibly excluding a significant population in remote areas. Moreover, a significant proportion of our patient population was derived from Regina as compared to Saskatoon despite comparable catchment areas and city populations. This disparity is likely attributable to the fact that Saskatoon’s patient population is much more heterogeneous including specialty cardiac cases that may have diluted the general HF population. In addition, our main definition of HF admissions to hospital was based on diagnostic codes instead of laboratory-confirmed cardiac injury findings and they were not retrospectively validated by a clinician. Next, we defined the influenza risk period as the day after the date of respiratory sampling, however, the infection onset date would have occurred earlier, which may have underestimated the true effect size. We also could not exclude the chance that some viruses isolated could represent incidental colonization rather than symptomatic infections, which would have biased our results towards the null. We were also unable to investigate the effect of co-infections separately.

## Conclusion

In conclusion, the incidence of HFHs during the influenza risk interval was 33 times higher than the control interval in patients with HF. The disparity in the incidence ratios was driven by the first three days following a positive influenza result. This robust finding supports the association between acute influenza infection and HFHs.

## Data accessibility statement

The datasets during and/or analyzed during the current study are available from the corresponding author on reasonable request.
